# Mapping the impact of malnutrition as defined by the Global Leadership Initiative on Malnutrition and nutrition impact symptoms on the possibility of returning to work after treatment for head and neck cancer

**DOI:** 10.1007/s00520-023-08252-x

**Published:** 2023-12-22

**Authors:** Sandra Einarsson, Anna Bokström, Göran Laurell, Ylva Tiblom Ehrsson

**Affiliations:** 1 Department of Food, Nutrition and Culinary Science, Umeå University, Umeå, Sweden; 2 Unit for Celiac Disease and Diabetes, Lund University, Department of Pediatrics, Skåne University Hospital, Malmö, Sweden; 3 Department of Surgical Sciences, Section of Otorhinolaryngology and Head & Neck Surgery, Uppsala University, Uppsala, Sweden

**Keywords:** Nutrition impact symptoms, GLIM, Return to work, Head neck cancer

## Abstract

**Purpose:**

This study aimed to investigate whether malnutrition or nutrition impact symptoms (NIS) affect the possibility of returning to work after treatment for head and neck cancer.

**Methods:**

Patients of working age with head and neck cancer were followed up from treatment initiation to 3 months (*n* = 238), 1 year (*n* = 182), and 2 years (*n* = 130) after treatment completion. The observed decrease in the number of patients over time was due to retirement, lack of follow-up, or death. Returning to work was dichotomised as yes or no. Malnutrition was diagnosed 7 weeks after treatment initiation using the Global Leadership Initiative on Malnutrition (GLIM) criteria. This time-point corresponds to the end of chemoradiotherapy or radiotherapy (with or without prior surgery), except for patients who underwent exclusive surgery. NIS were scored on a Likert scale (1–5) at each follow-up using the Head and Neck Patient Symptom Checklist^©^ (HNSC^©^). Nonparametric tests were used to analyse the ability of patients with/without malnutrition and high/low NIS scores to return to work.

**Results:**

At 3 months, 1 year, and 2 years after treatment completion, 135/238 (56.7%), 49/182 (26.9%), and 23/130 (17.7%) patients had not returned to work. Patients with malnutrition at 7 weeks after treatment initiation were more likely to not return to work at 3 months than those without malnutrition, 70.5% compared to 47.1% (p < 0.001). At all three follow-up time-points, patients reporting high scores for a number of NIS had more often not returned to work, with this pattern being most distinct at 2 years.

**Conclusion:**

Malnutrition according to the GLIM criteria at 7 weeks after treatment initiation and NIS assessed by the HNSC^©^ at subsequent follow-ups were predictors of the return-to-work process after treatment for up to 2 years.

**Trial registration number:**

ClinicalTrials.gov NCT03343236 (date of registration 17/11/2017).

**Supplementary Information:**

The online version contains supplementary material available at 10.1007/s00520-023-08252-x.

## Introduction

Currently, there are increased chances of surviving cancer due to more streamlined diagnostic procedures and treatment approaches [1]. Combined with the increased incidence rate of cancer, this has resulted in a higher number of cancer survivors. Surviving cancer may result in another life trajectory: a new identity [2] influenced by overarching contextual factors, including working ability [3]. Therefore, the ability to return to work (RTW) after cancer treatment is important [4, 5] but challenging; moreover, cancer survivors are more likely to be unemployed [6]. Compared with survivors of other cancer types, head and neck cancer (HNC) survivors have the highest risk of disability and quitting their job [7] or are less likely to RTW and be employed after treatment [8].

HNC is a malignancy of the upper aerodigestive tract, and most patients are diagnosed in their early 60 s [9]. However, the increase in human papillomavirus (HPV)-induced tumours has altered the demographics to include the younger population [10]. Treatment for HNC often comprise a multimodal approach of radiotherapy (RT), surgery, and/or medical treatment; further, it is intended to not only improve survival but also sustain function. However, HNC treatment can cause severe morbidity since several vital functions occur in the head and neck [11]. Acute toxicities result from damage to cells with rapid turnover and commonly develop during RT [12, 13]. Late toxicities and sequelae are caused by damage to cells with slow turnover and usually appear or progress months or even years after treatment termination. Regardless of the cells that are damaged, the toxicities and sequelae related to HNC treatment may affect different aspects of eating and drinking [14, 15]. These aspects can be defined as nutritional impact symptoms (NIS).

Frequently reported NIS due to HNC treatment include smell and taste alterations, pain, mucositis, dysphagia, xerostomia, and problems with teeth and chewing [9, 11, 16]. Physically, NIS may lead to reduced food intake, weight loss, reduced skeletal mass, and subsequent malnutrition. Psychologically, NIS related to food and eating may impose social constraints, with stigmatisation and withdrawal from social interactions during mealtimes. NIS also contribute to sick leaves among HNC survivors [17].

There is a need to elucidate the factors significantly related to the ability to RTW among individuals and in the society at large. Currently, more people of working age survive cancer; however, not all of them manage to fully RTW after treatment, with studies indicating that HNC survivors are especially vulnerable [7, 8]. Since many HNC survivors struggle with food and eating after treatment, identifying nutritional factors crucially involved in the RTW process may help healthcare professionals provide the support required to effectively facilitate RTW.

## Aim

This study aimed to investigate whether malnutrition according to the Global Leadership Initiative on Malnutrition (GLIM) criteria and NIS according to the Head and Neck Patient Symptom Checklist^©^ influence the ability to RTW after HNC treatment.

## Materials and methods

This study presents data on patients with HNC from an ongoing prospective observational study performed at three university hospitals in Sweden (ClinicalTrials.gov: NCT03343236). The patients were followed up through the trajectory of care for up to 2 years.

### Patients

**Fig. 1:**
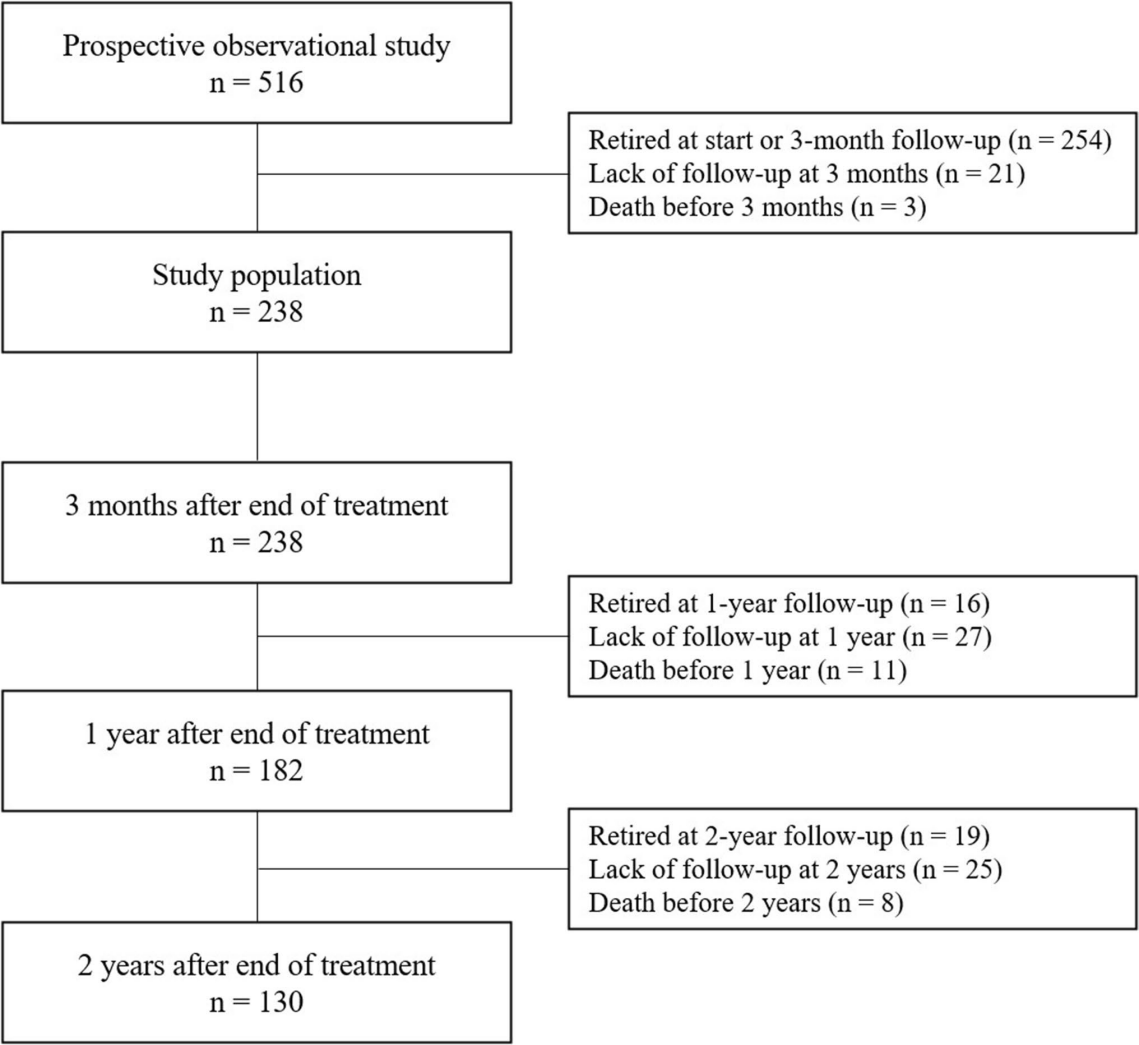
Number of patients with head and neck cancer in the present study. The number of patients decreased over time due to retirement, death, or lack of follow-up. Since this study presents data from an ongoing prospective observational study, patients without follow-up had not reached the time-point for that follow-up

Since recruitment began in 2015, 516 patients with HNC agreed to participate in the ongoing prospective observational study. The inclusion criteria were newly diagnosed HNC planned for curative treatment and a performance status of 0–2 according to the Eastern Cooperative Oncology Group Performance Status/World Health Organization (WHO) Performance Status [18]. The exclusion criteria were previous treatments for malignant neoplasms within the past 5 years (except skin cancer), inability to understand the Swedish language, severe alcohol abuse, and cognitive conditions. The reason for these exclusion criteria was the notion that they would have a profound impact on the ability to participate in the study and influence on study compliance. Further, in the present study, we excluded patients in full retirement (traditional or disabled) (Fig. 1). In Sweden, the mean age for retirement is 64.8 years (year 2022), the earliest an individual can draw pension is at the age of 63 years [19]. Almost half (254/516, 49.2%) of the patients enrolled in the prospective observational study had full retirement at the time of study recruitment or the 3-month follow-up. Additionally, in the present study, the number of patients decreased over time due to a lack of follow-up or death. Since this study presented data from an ongoing prospective observational study, patients without follow-up had not reached that follow-up time point.

### Data

Research nurses at three university hospitals collected data from the patients and medical records at treatment initiation; 7 weeks after treatment initiation; and 3 months, 1 year, and 2 years after the end of treatment. Due to the long travel distance, some measurements were performed at the local hospitals of patients.

We collected the following data: age, sex, tumour type (oropharynx, oral, larynx, other), tumour stage (I + II, III + IV), and treatment (RT ± surgery; surgery; chemoradiotherapy ± surgery; RT + other pharmacological treatment ± surgery; brachytherapy).

#### Working situation

Data on patients’ working situation was collected at the start of treatment and during the three follow-ups and was dichotomised into ‘returned to work’ (working full or part-time as planned) and ‘not returned to work’ (not working at all or working part-time due to partial sick leave, unemployment).

#### Malnutrition

GLIM is a global agreement that defines malnutrition by the combination of at least one phenotypic criterion with one etiological criterion [20]. The phenotypic criteria were defined as follows with GLIM reference in brackets: weight loss calculated by comparing self-reported weight at 6 months before treatment initiation with weight at treatment initiation, or weight at treatment initiation compared with weight at 7 weeks after treatment initiation (ref. > 5% within 6 months, or > 10% beyond 6 months); BMI < 20 kg/m^2^ if < 70 years, or < 22 kg/m^2^ if > 70 years (ref. < 20 kg/m^2^ if < 70 years, or < 22 kg/m^2^ if > 70 years); Fat-free mass index, FFMI calculated as FFM divided by the square of height, FFM/m^2^, < 17 kg/m^2^ for males and < 15 kg/m^2^ for females (ref. reduced by validated body composition techniques). Body composition parameters were obtained using a bioelectrical impedance analysis (BIA) device (BC418MA, Tanita Corporation, Tokyo, Japan). The BIA measurements were conducted by a small group of trained staff, where research nurses at each location had the main responsibility of securing its accuracy. Patients were weighed without outdoor clothing, socks, or shoes. For each measurement, 1 kg was deducted for clothing. The BIA device was only available at university hospitals; accordingly, due to logistical reasons, some participants underwent measurements using a regular body weight scale (not BIA device) at their local hospital leading to missing FFMI values. In case of missing FFMI values, patients were diagnosed with malnutrition if they had another combination of phenotypic (weight loss or low BMI) and etiological criteria. If patients lacked any other GLIM criteria, they were reported to have missing values.

The phenotypic criteria were defined as follows with GLIM reference in brackets: partial or no food intake with the need for artificial nutrition (ref. ≤ 50% of energy requirement > 1 week, or any reduction for > 2 weeks); C-reactive protein > 5 mg/L (ref. acute disease/injury or chronic disease-related). Participants answered questions about their oral intake and the use of tube feeding and/or parenteral nutrition. Blood samples were collected for C-reactive protein analysis.

The present study is the next in line of a series of publications made by the research group for mapping malnutrition in patients with HNC according to GLIM [21–23]. As indicated in our previous studies, the highest frequency of malnutrition was observed at 7 weeks after treatment initiation (corresponding to the end of treatment for patients being treated with single-modality RT or chemoradiotherapy), with very few patients being diagnosed with malnutrition at subsequent follow-ups. Therefore, the main measurement point of malnutrition was set at 7 weeks after treatment initiation, and therefore, data for all GLIM criteria were used from that time point. In addition, for a base-line reference value, malnutrition according to GLIM was assessed at the start of treatment.

#### Nutrition Impact Symptoms

Regarding NIS, each participant digitally completed the Head and Neck Patient Symptom Checklist^©^ (HNSC^©^) [24] at treatment initiation and follow-ups (3 months, 1 year, and 2 years after the end of treatment). Some patients completed the HNSC^©^ on paper and their answers were subsequently transferred into the web-based program by the research nurses. The instrument includes 17 NIS commonly experienced by patients with HNC and has been validated to determine symptoms that interfere with their dietary intake [24, 25]. Moreover, the instrument has an additional line where the patient can write free-text answers for non-listed symptoms.

HNSC^©^ has two parts related to nutrition intake, which are both rated on a five-point Likert scale (1 = not at all, 5 = a lot). The first part measures the intensity of the symptom, i.e., the frequency of the symptom during the last 3 days, while the second part measures the interference of the symptom with oral intake, i.e., if the symptom affects the ability to eat. If a patient chose ‘1 = not at all’ as the intensity of one symptom, they did not proceed to the second part regarding the interference of that symptom. We investigated the intensity of NIS experienced by each patient and their interference with oral intake using the HNSC^©^ five-point Likert scale. In addition, number of NIS experienced by each patient at different time points was defined from intensity score 2–5 on the Likert scale.

### Statistical analyses

Statistical analyses were performed using IBM SPSS version 28.0. Age and BMI at treatment initiation are presented as mean (± standard deviation). Number (%) was used for patient characteristics at treatment initiation and NIS at different time-points (treatment initation as well as 3 months, 1 year, and 2 years after treatment termination). The Chi-squared test was used to analyse differences between patients with and without malnutrition according to the GLIM criteria at 7 weeks after treatment initiation and their ability to RTW at the three follow-up points. The Chi-squared test was also used to analyse differences between patients with and without tube feeding at 7 weeks after treatment initiation and their ability to RTW at the 3-month follow-up. The Friedman test was used to test the change in NIS over time. The Mann–Whitney U-test was used to compare the number of NIS at different follow-up points between patients with and without malnutrition at 7 weeks after treatment initiation. Additionally, the Mann–Whitney U-test was used to analyse whether NIS scores (1–5) had an impact on the ability to RTW. Statistical significance was set at p < 0.05. Exact p-values are given for smaller groups instead of asymptotic p-values.

## Results

### Patient characteristics and working situation

There were in total 238 patients from start. The mean age was 55.1 years (± 9.2); further, the majority of participants were men (138/238, 70.6%). Oropharynx was the most common tumour site (119/238, 50.0%). In addition, 62/238 patients (26.1%) had tumour of the oral cavity and 18/238 patients (7.6%) tumour of the larynx. Other tumour sites were as follows: hypopharynx; nasopharynx; salivary gland cancer; nasal and sinus cancer; cancer of the external auditory canal; ear cancer; or cancer of unknown primary (39/238, 16.4% in total). In the study population, 153/238 patients (64.3%) had stage I or II tumours and 83/238 patients (34,9%) had stage III or IV tumours, according to the Union for International Cancer Controlʹs (UICC) 8. Stage was not applicable in two patients. The treatment approaches were: single modality RT (85/238, 35.7%); surgery (25/238, 10.5%); surgery commencing RT (43/238, 18.1%), chemoradiotherapy (72/238, 30.3%); surgery commencing chemoradiotherapy (10/238, 4.2%); or brachytherapy (3/238, 1.3%). Measurement time points (start of treatment, 7 weeks after the start of treatment, 3 months, 1 year, and 2 years after end of treatment) for each treatment modality are shown in Table 1. At treatment initiation, 65/238 (27.3%) patients were working full or part time as planned whereas 173/238 (72.7%) were not working at all or were working part time because of sick leave or unemployment. At 3 months after treatment completion, more than half (135/238, 56.7%) of the patients did not RTW. Comparatively, proportionally fewer patients did not RTW at the 1-year (49/182, 26.9%) and 2-year (23/130, 17.7%) follow-ups.

**Table 1:** Measurement points for each treatment modality in patients with head and neck cancer

		Treatment start	7-week follow-up	3-month follow-up	1-year follow-up	2-year follow-up
RT	*n* = 85	First day of RT	Last day of RT	Last day of RT –3 months	Last day of RT –1 year	Last day of RT –2 years
CRT	*n* = 72	First day of RT	Last day of RT	Last day of RT –3 months	Last day of RT –1 year	Last day of RT –2 years
Surgery	*n* = 25	Day of surgery	Day of surgery –7 weeks	7-week follow-up –3 months	7-week follow-up –1 year	7-week follow-up –2 years
Surgery before RT	*n* = 43	Day of surgery	Last day of RT	Last day of RT –3 months	Last day of RT –1 year	Last day of RT –2 years
Surgery before CRT	*n* = 10	Day of surgery	Last day of CRT	Last day of CRT –3 months	Last day of CRT –1 year	Last day of CRT – 2years
Brachytherapy	*n* = 3	Day of treatment	Day of treatment –7 weeks	7-week follow-up –3 months	7-week follow-up –1 year	7-week follow-up –2 years

Radiotherapy (RT), Chemoradiotherapy (CRT)

### Malnutrition at 7 weeks and return to work

The overall mean BMI at treatment initiation was 27.7 (± 5.0) kg/m^2^ and 17/238 (7.1%) patients were malnourished according to the GLIM criteria (missing, *n* = 7). At 7 weeks after treatment initiation, 88/238 (37.0%) patients were malnourished according to the GLIM criteria (missing, *n* = 12). Patients with malnutrition were more likely to not RTW at 3 months after treatment completion than patients without malnutrition (p < 0.001). Among the 88 patients with malnutrition, 62 (70.5%) patients did not RTW at 3 months compared with the 65 (47.1%) patients who were not malnourished. Having tube feeding at 7 weeks after the start of treatment did not interfere with the possibility to RTW at 3 months (*p* = 0.062). At 7 weeks, 58 patients had partial or full tube feeding and seven of those had not RTW at 3 months. Fifteen patients had tube feeding at 3 months, and one of those had not RTW at that time point. No difference was observed between patients with malnutrition or no malnutrition at 7 weeks and the possibility to RTW at 1 year (*p* = 0.362) or 2 years (*p* = 0.384) after treatment completion.

### Nutrition impact symptoms at different time-points

Regarding NIS intensity, ‘dry mouth’ was the symptom that received the highest score (5 = a lot) on the Likert scale from most patients at 3 months (50/238, 21.0%), 1 year (22/182, 12.1%), and 2 years (15/130, 11.5%) after treatment termination (Table 2). Regarding NIS interference, ‘taste changes’ was the symptom that received the highest score (5 = a lot) on the Likert scale from most patients at 3 months (22/238, 9.2%), followed by ‘dry mouth’ (21/238, 8.8%) (Table 3). A few patients gave the highest interference score at the 1-year and 2-year follow-ups. Overall, the scoring of intensity and interference with oral intake of the NIS from the HNSC^©^ were lower at subsequent follow-ups for most of the symptoms (Table 2 and 3). Most NIS received significantly lower scores from patients over time (Appendix, Tables A-B).

**Table 2:** Number of patients (%) at different time-points (3 months, 1 year, and 2 years after treatment completion) that assessed nutrition impact symptoms (NIS) on a five-point Likert scale (1 = not at all, 5 = a lot) using the Head and Neck Patient Symptom Checklist^©^ (HNSC^©^) [24]. The first part of the HNSC^©^ is presented and shows NIS **intensity,** which assesses the frequency of the symptom during the last 3 days

	3 months (*n* = 238)						1 year (*n* = 182)						2 years (*n* = 130)					
	*Likert scale*						*Likert scale*						*Likert scale*					
NIS	1	2	3	4	5	Missing	1	2	3	4	5	Missing	1	2	3	4	5	Missing
Pain	92 (38,7)	61 (25.6)	27 (11.3)	32 (13.4)	9 (3.8)	17 (7.1)	99 (54.4)	38 (20.9)	18 (9.9)	9 (4.9)	5 (2.7)	13 (7.1)	83 (63.8)	18 (13.8)	10 (7.7)	3 (2.3)	2 (1.5)	14 (10.8)
Anxious	88 (37.0)	67 (28.2)	31 (13.0)	28 (11.8)	7 (2.9)	17 (7.1)	82 (45.1)	43 (23.6)	30 (16.5)	8 (4.4)	4 (2.2)	15 (8.2)	64 (49.2)	30 (23.1)	15 (11.5)	6 (4.6)	1 (0.8)	14 (10.8)
Dry mouth	23 (9.7)	30 (12.6)	46 (19.3)	72 (30.3)	50 (21.0)	17 (7.1)	22 (12.1)	42 (23.1)	34 (18.7)	47 (25.8)	22 (12.1)	15 (8.2)	26 (20.0)	27 (20.8)	19 (14.6)	29 (22.3)	15 (11.5)	14 (10.8)
Loss of appetite	82 (34.5)	47 (19.7)	37 (15.5)	39 (16.4)	15 (6.3)	18 (7.6)	96 (52.7)	43 (23.6)	19 (10.4)	9 (4.9)	1 (0.5)	14 (7.7)	83 (63.8)	19 (14.6)	9 (6.9)	4 (3.1)	1 (0.8)	14 (10.8)
Constipation	164 (68.9)	35 (14.7)	13 (5.5)	6 (2.5)	4 (1.7)	16 (6.7)	130 (71.4)	25 (13.7)	10 (5.5)	4 (2.2)	0 (0.0)	13 (7.1)	95 (73.1)	10 (7.7)	7 (5.4)	3 (2.3)	0 (0.0)	15 (11.5)
Feeling full	86 (36.1)	49 (20.6)	44 (18.5)	37 (15.5)	5 (2.1)	17 (7.1)	85 (46.7)	42 (23.1)	23 (12.6)	17 (9.3)	2 (1.1)	13 (7.1)	64 (49.2)	21 (16.2)	18 (13.8)	8 (6.2)	3 (2.3)	16 (12.3)
Depressed	130 (54.6)	47 (19.7)	30 (12.6)	11 (4.6)	4 (1.7)	16 (6.7)	114 (62.6)	32 (17.6)	15 (8.2)	7 (3.8)	0 (0.0)	14 (7.7)	80 (61.5)	23 (17.7)	7 (5.4)	6 (4.6)	0 (0.0)	14 (10.8)
Thick saliva	71 (29.8)	66 (27.7)	33 (13.9)	39 (16.4)	13 (5.5)	16 (6.7)	56 (30.8)	42 (23.1)	37 (20.3)	24 (13.2)	7 (3.8)	16 (8.8)	48 (36.9)	33 (25.4)	15 (11.5)	14 (10.8)	6 (4.6)	14 (10.8)
Diarrhoea	177 (74.4)	27 (11.3)	11 (4.6)	5 (2.1)	2 (0.8)	16 (6.7)	144 (79.1)	13 (7.1)	6 (3.3)	5 (2.7)	1 (0.5)	13 (7.1)	94 (72.3)	14 (10.8)	3 (2.3)	3 (2.3)	2 (1.5)	14 (10.8)
Sore mouth	102 (42.9)	65 (27.3)	25 (10.5)	23 (9.7)	7 (2.9)	16 (6.7)	105 (57.7)	24 (13.2)	23 (12.6)	14 (7.7)	3 (1.6)	13 (7.1)	74 (56.9)	27 (20.8)	8 (6.2)	6 (4.6)	1 (0.8)	14 (10.8)
Lack of energy	65 (27.3)	63 (26.5)	43 (18.1)	39 (16.4)	11 (4.6)	17 (7.1)	72 (39.6)	51 (28.0)	18 (9.9)	23 (12.6)	4 (2.2)	14 (7.7)	53 (40.8)	30 (23.1)	18 (13.8)	11 (8.5)	4 (3.1)	14 (10.8)
Nausea	185 (77.7)	26 (10.9)	7 (2.9)	2 (0.8)	2 (0.8)	16 (6.7)	143 (78.6)	17 (9.3)	5 (2.7)	4 (2.2)	0 (0.0)	13 (7.1)	101 (77.7)	9 (6.9)	3 (2.3)	3 (2.3)	0 (0.0)	14 (10.8)
Diff. chewing	128 (53.8)	44 (18.5)	18 (7.6)	24 (10.1)	7 (2.9)	17 (7.1)	109 (59.9)	32 (17.6)	17 (9.3)	8 (4.4)	2 (1.1)	14 (7.7)	84 (64.6)	18 (13.8)	7 (5.4)	2 (1.5)	4 (3.1)	15 (11.5)
Smells bother me	168 (70.6)	26 (10.9)	13 (5.5)	6 (2.5)	8 (3.4)	17 (7.1)	133 (73.1)	14 (7.7)	12 (6.6)	7 (3.8)	3 (1.6)	13 (7.1)	89 (68.5)	15 (11.5)	8 (6.2)	3 (2.3)	1 (0.8)	14 (10.8)
Vomiting	201 (84.5)	16 (< 6.7)	0 (0.0)	3 (1.3)	2 (0.8)	16 (6.7)	157 (86.3)	6 (3.3)	5 (2.7)	0 (0.0)	0 (0.0)	14 (7.7)	108 (83.1)	4 (3.1)	3 (2.3)	1 (0.8)	0 (0.0)	14 (10.8)
Diff. swallowing	104 (43.7)	55 (23.1)	28 (11.8)	23 (9.7)	11 (4.6)	17 (7.1)	90 (49.5)	37 (20.3)	22 (12.1)	15 (8.2)	3 (1.6)	15 (8.2)	61 (46.9)	35 (26.9)	10 (7.7)	7 (5.4)	3 (2.3)	14 (10.8)
Taste changes	38 (16.9)	54 (22.7)	39 (16.4)	54 (22.7)	37 (15.5)	16 (6.7)	53 (29.1)	41 (22.5)	24 (13.2)	38 (20.9)	13 (7.1)	13 (7.1)	54 (41.5)	21 (16.2)	12 (9.2)	21 (16.2)	8 (6.2)	14 (10.8)
Other: Specify^1^	100 (42.0)	5 (2.1)	13 (5.5)	15 (6.3)	11 (4.6)	94 (39.5)	86 (47.3)	6 (3.3)	6 (3.3)	8 (4.4)	7 (3.8)	69 (37.9)	61 (46.9)	4 (3.1)	2 (1.5)	5 (3.8)	4 (3.1)	54 (41.5)

^1^Patients could express other NIS symptoms not outlined in the HNSC^©^ form

**Table 3:** Number of patients (%) at different time-points (3 months, 1 year, and 2 years after treatment completion) that assessed nutrition impact symptoms (NIS) on a five-point Likert scale (1 = not at all, 5 = a lot) using the Head and Neck Patient Symptom Checklist^©^ (HNSC^©^) [24]. The second part of the HNSC ^©^ is presented and shows NIS **interference,** which assesses the interference of the symptom with oral intake

	3 months (*n* = 238)							1 year (*n* = 182)							2 years (*n* = 130)						
	*Likert scale*							*Likert scale*							*Likert scale*						
NIS	1	2	3	4	5	Part I*	Missing	1	2	3	4	5	Part I*	Missing	1	2	3	4	5	Part I*	Missing
Pain	30 (12.6)	34 (14.3)	35 (14.7)	18 (7.6)	10 (4.2)	92 (38,7)	19 (8.0)	25 (13.7)	19 (10.4)	16 (8.8)	8 (4.4)	1 (0.5)	99 (54.4)	14 (7.7)	14 (10.8)	9 (6.9)	5 (3.8)	4 (3.1)	0 (0.0)	83 (63.8)	15 (11.5)
Anxious	69 (29.0)	34 (14.3)	18 (7.6)	7 (2.9)	3 (1.3)	88 (37.0)	19 (8.0)	45 (24.7)	23 (12.6)	11 (6.0)	2 (1.1)	0 (0.0)	82 (45.1)	19 (10.4)	31 (23.8)	11 (8.5)	7 (5.4)	1 (0.8)	0 (0.0)	64 (49.2)	16 (12.3)
Dry mouth	52 (21.8)	33 (13.9)	44 (18.5)	45 (18.9)	21 (8.8)	23 (9.7)	20 (8.4)	51 (28.0)	32 (17.6)	30 (16.5)	24 (13.2)	3 (1.6)	22 (12.1)	20 (11.0)	32 (24.6)	28 (21.5)	17 (13.1)	11 (8.5)	1 (0.8)	26 (20.0)	15 (11.5)
Loss of appetite	9 (3.8)	41 (17.2)	42 (17.6)	32 (13.4)	12 (5.0)	82 (34.5)	20 (8.4)	5 (2.7)	38 (20.9)	21 (11.5)	5 (2.7)	1 (0.5)	96 (52.7)	16 (8.8)	4 (3.1)	17 (13.1)	8 (6.2)	4 (3.1)	0 (0.0)	83 (63.8)	14 (10.8)
Constipation	33 (13.9)	12 (5.0)	11 (4.6)	1 (0.4)	0 (0.0)	164 (68.9)	17 (7.1)	23 (12.6)	13 (7.1)	2 (1.1)	0 (0.0)	0 (0.0)	130 (71.4)	14 (7.7)	7 (5.4)	10 (7.7)	1 (0.8)	2 (1.5)	0 (0.0)	95 (73.1)	15 (11.5)
Feeling full	38 (16.0)	44 (18.5)	37 (15.5)	12 (5.0)	2 (0.8)	86 (36.1)	19 (8.0)	22 (12.1)	36 (19.8)	18 (9.9)	6 (3.3)	1 (0.5)	85 (46.7)	14 (7.7)	24 (18.5)	15 (11.5)	6 (4.6)	4 (3.1)	0 (0.0)	64 (49.2)	17 (13.1)
Depressed	36 (15.1)	29 (12.2)	19 (8.0)	6 (2.5)	1 (0.4)	130 (54.6)	17 (7.1)	24 (13.2)	15 (8.2)	10 (5.5)	4 (2.2)	0 (0.0)	114 (62.6)	15 (8.2)	20 (15.4)	10 (7.7)	4 (3.1)	1 (0.8)	1 (0.8)	80 (61.5)	14 (10.8)
Thick saliva	64 (26.9)	32 (13.4)	31 (13.0)	17 (7.1)	6 (2.5)	71 (29.8)	17 (7.1)	54 (29.7)	32 (17.6)	14 (7.7)	9 (4.9)	1 (0.5)	56 (30.8)	16 (8.8)	40 (30.8)	17 (13.1)	8 (6.2)	2 (1.5)	1 (0.8)	48 (36.9)	14 (10.8)
Diarrhoea	31 (13.0)	10 (4.2)	2 (0.8)	1 (0.4)	0 (0.0)	177 (74.4)	17 (7.1)	19 (10.4)	2 (1.1)	4 (2.2)	0 (0.0)	0 (0.0)	144 (79.1)	13 (7.1)	14 (10.8)	6 (4.6)	1 (0.8)	1 (0.8)	0 (0.0)	94 (72.3)	14 (10.8)
Sore mouth	29 (12.2)	41 (17.2)	25 (10.5)	18 (7.6)	6 (2.5)	102 (42.9)	17 (7.1)	16 (8.8)	23 (12.6)	18 (9.9)	4 (2.2)	2 (1.1)	105 (57.7)	14 (7.7)	22 (16.9)	8 (6.2)	6 (4.6)	3 (2.3)	3 (2.3)	74 (56.9)	14 (10.8)
Lack of energy	76 (31.9)	43 (18.1)	25 (10.5)	8 (3.4)	3 (1.3)	65 (27.3)	18 (7.6)	57 (31.3)	28 (15.4)	8 (4.4)	3 (1.6)	0 (0.0)	72 (39.6)	14 (7.7)	41 (31.5)	17 (13.1)	3 (2.3)	2 (1,5)	0 (0.0)	53 (40.8)	14 (10.8)
Nausea	8 (3.4)	20 (8.4)	6 (2.5)	3 (1.3)	0 (0.0)	185 (77.7)	16 (6.7)	10 (5.5)	10 (5.5)	5 (2.7)	1 (0.5)	0 (0.0)	143 (78.6)	13 (7.1)	4 (3.1)	8 (6.2)	1 (0.8)	1 (0.8)	1 (0.8)	101 (77.7)	14 (10.8)
Diff. chewing	16 (6.7)	27 (11.3)	25 (10.5)	15 (6.3)	9 (3.8)	128 (53.8)	18 (7.6)	11 (6.0)	24 (13.2)	16 (8.8)	6 (3.3)	2 (1.1)	109 (59.9)	14 (7.7)	8 (6.2)	10 (7.7)	7 (5.4)	2 (1.5)	4 (3.1)	84 (64.6)	15 (11.5)
Smells bother me	13 (5.5)	25 (10.5)	6 (2.5)	5 (2.1)	3 (1.3)	168 (70.6)	18 (7.6)	15 (8.2)	8 (4.4)	9 (4.9)	3 (1.6)	1 (0.5)	133 (73.1)	13 (7.1)	14 (10.8)	5 (3.8)	6 (4.6)	2 (1.5)	0 (0.0)	89 (68.5)	14 (10.8)
Vomiting	9 (3.8)	9 (3.8)	1 (0.4)	2 (0.8)	0 (0.0)	201 (84.5)	16 (6.7)	6 (3.3)	4 (2.2)	0 (0.0)	0 (0.0)	0 (0.0)	157 (86.3)	15 (8.2)	3 (2.3)	2 (1.5)	2 (1.5)	1 (0.8)	0 (0.0)	108 (83.1)	14 (10.8)
Diff. swallowing	18 (7.6)	39 (16.4)	24 (10.1)	23 (9.7)	11 (4.6)	104 (43.7)	19 (8.0)	14 (7.7)	36 (19.8)	16 (8.8)	9 (4.9)	2 (1.1)	90 (49.5)	15 (8.2)	11 (8.5)	23 (17.7)	11 (8.5)	7 (5.4)	3 (2.3)	61 (46.9)	14 (10.8)
Taste changes	33 (13.9)	55 (23.1)	36 (15.1)	37 (15.5)	22 (9.2)	38 (16.9)	17 (7.1)	31 (17.0)	30 (16.5)	35 (19.2)	15 (8.2)	5 (2.7)	53 (29.1)	13 (7.1)	25 (19.2)	18 (13.8)	12 (9.2)	6 (4.6)	1 (0.8)	54 (41.5)	14 (10.8)
Other: Specify^1^	14 (5.9)	6 (2.5)	7 (2.9)	11 (4.6)	5 (2.1)	100 (42.0)	95 (39.9)	11 (6.0)	5 (2.7)	6 (3.3)	3 (1.6)	1 (0.5)	86 (47.3)	70 (38.5)	9 (6.9)	3 (2.3)	0 (0.0)	1 (0.8)	2 (1.5)	61 (46.9)	54 (41.5)

^1^Patients could express other NIS symptoms not outlined in the HNSC^©^ form

*Intensity Score 1 in the first part of the HNSC form meant that patients did not fill in the second part of the HNSC^©^ form

Number of NIS that each patient experienced at different time-points were defined from values of 2–5 on the Likert scale for the intensity score. At 3 months, 1 year, and 2 years, the highest number of NIS experienced by individual patients was eight (31/238 [13.0%] patients), ten (17/182 [9.3%] patients), and six (15/130 [11.5%] patients), respectively. Patients with malnutrition at 7 weeks after treatment initiation experienced a significantly higher number of NIS at the 3-month (*p* = 0.001) and 1-year (*p* = 0.015) follow-ups, respectively, compared with patients without malnutrition. There was no significant difference in the number of NIS at 2 years between patients with and without malnutrition at 7 weeks (*p* = 0.151).

### Nutrition impact symptoms and return to work

**Fig. 2:**
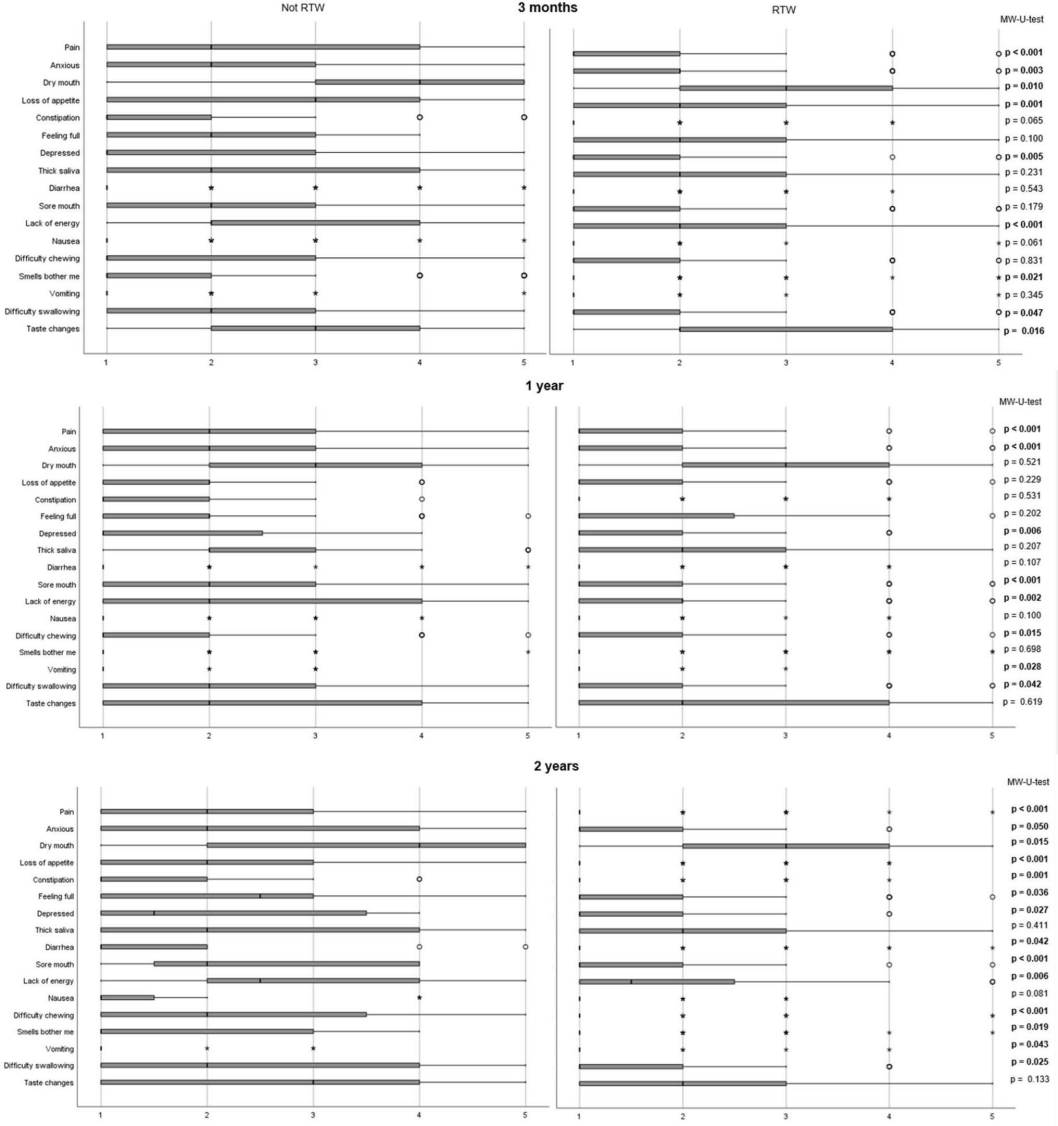
Boxplots (median, Q1, Q3, min, max) on **intensity** of nutrition impact symptoms (NIS) divided on the ability to return to work (RTW) at 3 months, 1 year, and 2 years after treatment completion for head and neck cancer. NIS were scored on a five-point Likert scale (1 = not at all, 5 = a lot) using the Head and Neck Patient Symptom Checklist^©^ (HNSC^©^) [24]. Intensity assesses the frequency of the symptom during the last 3 days. Significant p-values are shown in bold

**Fig. 3:**
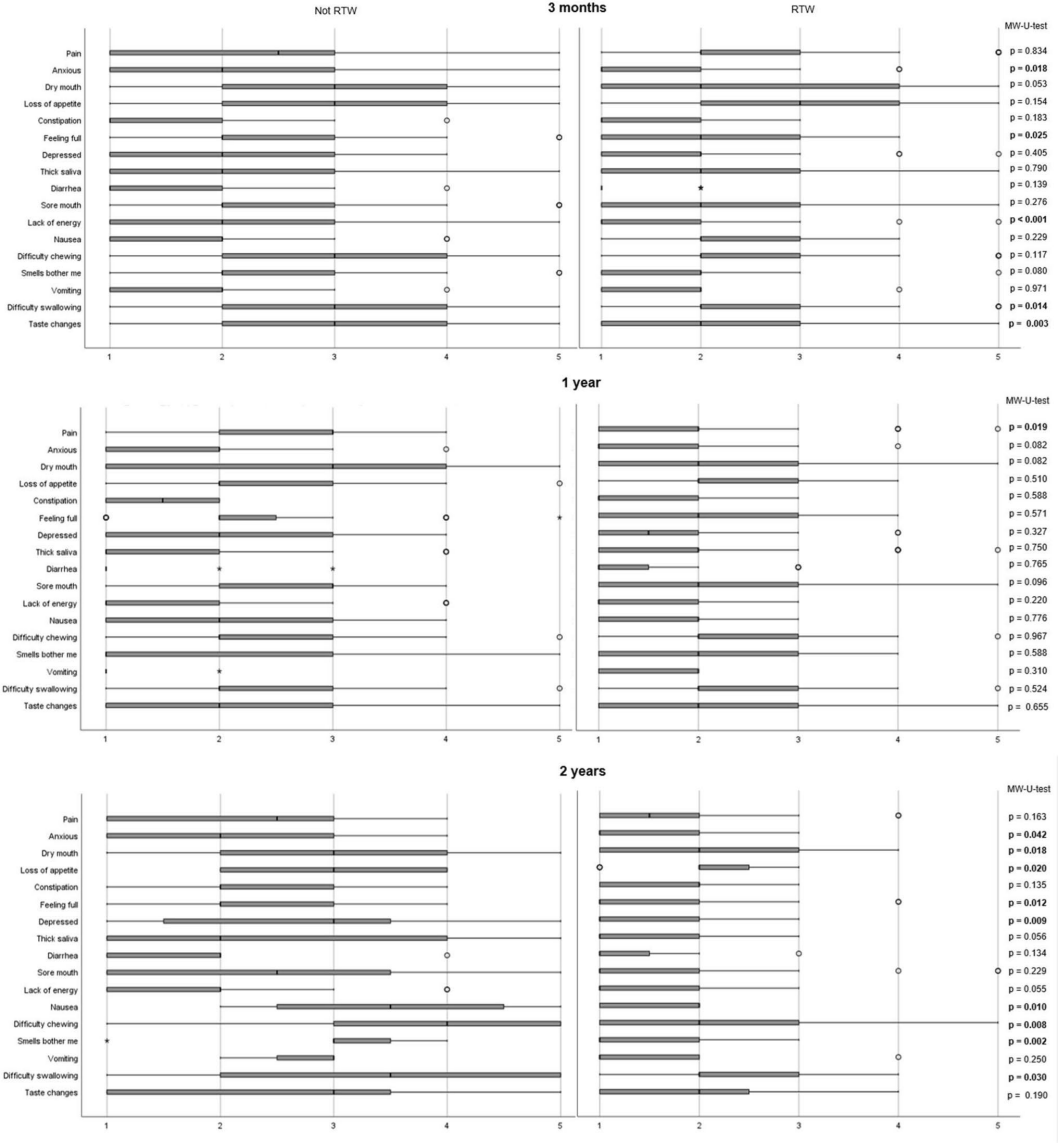
Boxplots (median, Q1, Q3, min, max) on **interference** of nutrition impact symptoms (NIS) divided on the ability to return to work (RTW) at 3 months, 1 year, and 2 years after treatment completion for head and neck cancer. NIS were scored on a five-point Likert scale (1 = not at all, 5 = a lot) using the Head and Neck Patient Symptom Checklist^©^ (HNSC^©^) [24]. Interference assesses the interference of the symptom with oral intake. Significant p-values are shown in bold. The exact p-value is given for smaller groups instead of the asymptotic *p*-value

At the three follow-ups (3 months, 1 year, and 2 years), patients who did not RTW reported significantly higher scores on the Likert scale for both intensity and interference of a number of NIS compared with patients who could RTW (Figs. 2 and 3), with this difference being most distinct at the 2-year follow-up. Patients who did not RTW at 2 years reported significantly higher scores on the Likert scale for 14 and 9 NIS with respect to intensity and interference, respectively, compared with patients who could RTW.

## Discussion

Our findings showed that malnutrition according to the GLIM criteria at 7 weeks after treatment initiation and NIS at subsequent follow-ups were predictors of RTW for up to 2 years after treatment termination. Less than half of the HNC survivors could RTW at 3 months after treatment termination. Since this study presented data from an ongoing prospective observational study, there was a gradual loss of patients to follow-up during the study period. Nonetheless, at subsequent follow-ups, there was an increase in the proportion of patients who could RTW, with only 17.7% of patients at the 2-year follow-up being unable to RTW. Given the increasing number of cancer survivors, there has been growing interest in elucidating the factors that promote and impede the RTW process. A systematic review conducted by Morales et al. [26] addressed different clinical, physical, psychological, emotional, and social factors as well as employment- and work-related factors that could facilitate the RTW process in patients treated for HNC. Managing RTW is a multifaceted process in which nutrition is among the crucial aspects. Our findings significantly contribute to the literature given the wide range of NIS examined and the long-term follow-up period (up to 2 years). Additionally, to our knowledge, there has been no previous study on malnutrition according to the GLIM criteria and its impact on the ability to RTW after treatment for HNC.

In patients with HNC, malnutrition often develops during the last treatment phase [21] and seldom appears or progresses after that time point in patients with loco-regional control [22]. Therefore, we wanted to explore if and for how long malnutrition at 7 weeks after treatment initiation impacts the ability to RTW. Compared with patients without malnutrition, patients with malnutrition were significantly more likely to not RTW at 3 months after treatment termination; however, a similar pattern was not observed at the 1-year and 2-year follow-ups. This suggests that treatment-related malnutrition only impacts the possibility of RTW during the first months after treatment termination. Malnutrition in HNC survivors is caused by a combination of inflammation and reduced food intake [21]. Inflammation-triggering factors are most likely linked to both cancer and its treatment [27–29], which result in the breakdown of important tissues, including muscle tissue [30]. The consequences of malnutrition and loss of muscle mass are multifaceted; nonetheless, reduced strength and function as well as delayed recovery after disease [31] could be considered as crucial factors hindering the RTW process. Accordingly, early interventions for preserving muscle mass are crucial [32] through a multimodal approach combining nutrition therapy with exercise [33]. This could help prevent the development of malnutrition rather than manage it upon establishment. The European Society for Clinical Nutrition and Metabolism, ESPEN, recommends nutrition therapy, which is characterized by individualised nutritional counselling and/or oral nutritional supplements, to minimise nutritional deterioration during RT [34]. Nutrition therapy may be delayed by the assumption that patients with malnutrition are underweight, which may increase the risk of malnutrition in patients with normal or even higher BMI are overlooked by healthcare professionals. Malnutrition and low muscle mass are often masked by normal weight or obesity [33]. In our study, the overall mean BMI at treatment initiation corresponded to pre-obesity, according to the BMI cutoffs declared by the WHO [35]. In industrialised countries such as Sweden, patients with HNC are often overweight throughout the trajectory of care [36–38]. Therefore, it is important for healthcare professionals to use appropriate methods to objectively assess patients’ nutritional status. The GLIM criteria address a wide range of etiologic factors that cause malnutrition, regardless of the BMI [20]. Repeated screening for malnutrition during treatment regardless of the BMI and using the GLIM criteria for diagnosis could crucially contribute towards identifying patients who require proactive multimodal interventions that preserve muscle mass and reduce the risk of malnutrition, which may facilitate RTW following termination of HNC treatment.

To further explore the relationship between nutritional status and RTW among HNC survivors, we examined the longitudinal development of NIS. We found that HNC survivors experienced different NIS even at the final 2-year follow-up; additionally, patients with higher scores for both intensity and interference of a number of NIS were less likely to RTW, especially at the 2-year follow up. This indicates that although the proportion of HNC survivors who RTW increased over time, some HNC survivors still struggled long term after treatment. The experience following HNC treatment has been described as tedious [15, 39], with some HNC survivors reporting deterioration, rather than improvement, in the long-term health-related quality of life (HRQoL) [40]. This impairment of HRQoL is mainly attributed to the remaining treatment sequelae [23, 40], which cause eating problems that substantially impede daily life activities [41, 42]. NIS following HNC treatment can lead to avoidance of eating with other people [41, 42] and in public spaces [42]. For example, Crowder et al. [43] described the psychosocial burden of chronic NIS and how it markedly affected the day-to-day lives of HNC survivors. Therefore, NIS could impede the ability to RTW since work involves numerous social contexts, including eating together with others. Further, the present study showed that many HNC survivors did not experience a single NIS. Given the complexity of the situation, several aspects that are crucial for the well-being of the patient are often lost in the information transfer between the patient and healthcare professionals, leading to unmet needs [44]. Our study showed that the HNSC^©^ is a useful tool for identifying the symptom burden affecting oral intake in HNC survivors, and thus can reveal important aspects regarding the RTW process. Using the HNSC^©^ to assess NIS and their longitudinal progression, as well as specifically asking how they impact the ability to RTW, can increase awareness among healthcare professionals regarding patients’ issues, which might inform early and individualised nutrition therapy as well as provision of support during the trajectory of care. Taken together, the HNSC^©^ can be used to assess patients not able to RTW, and therefore is an applicable instrument in the rehabilitation process.

This study has several strengths, including its large sample size, regular follow-ups, and extensive data collection. The study sample aligns with the general HNC population with respect to sex, tumour type, and treatment type; [9] however, two aspects of the study population are important. First, we only included patients with a WHO performance status of 0–2, which represents patients with better general conditions at baseline. In addition, exclusion criteria for study participation were severe alcohol abuse and declined cognitive condition. Therefore, our study sample most likely had a better nutritional status than the HNC population in general. Second, patients were lost to follow-up over time because of death or lack of follow-up. The measurements were performed by trained staff. Due to patient travelling logistics in relation to the BIA assessments, the time point for assessment could vary during the day and it was not possible to secure a fasting morning measurement for all patients. Furthermore, the RTW process is complex and multifaceted [26]. The tumour site, treatment type, HRQoL, appearance, and employment factors are crucial for the RTW process [17, 45, 46]. Therefore, it is difficult to differentiate between the direct and indirect effects of nutritional factors. Future studies are warranted to examine the impact of rehabilitation, with a focus on nutritional therapy, on a patient’s ability to RTW after HNC treatment. In addition, the present study did not seek to address how the aetiology of the disease and disease burden might affect the RTW process. There is an increasing interest in how HPV-induced tumours may affect nutritional status with the theory that the proinflammatory cytokine secretion associated with HPV-infection has the potential to generate nutritional disorders e.g., sarcopenia [47]. With the growing number of HPV-positive HNC tumours, this is a research area with great potential.

In conclusion, in patients with HNC, malnutrition according to the GLIM criteria at 7 weeks after treatment initiation and NIS assessed by the HNSC^©^ at subsequent follow-ups were predictors of the RTW process after treatment for up to 2 years. HNC survivors require extra attention to allow them to RTW after treatment; moreover, the RTW process is multifaceted and nutrition is among the key aspects. Using the GLIM criteria for the diagnosis procedure during treatment can help healthcare professionals identify patients requiring proactive multimodal interventions, including nutrition therapy. Using the HNSC^©^ to longitudinally assess NIS can facilitate individualised nutrition therapy and support, especially for patients struggling with the RTW process from a long-term perspective.

### Supplementary Information

Below is the link to the electronic supplementary material.

Supplementary file1 (DOCX 22 KB)

## Data Availability

The datasets are available from the corresponding author upon reasonable request.
