# Supplementary material for: Mapping the impact of malnutrition as defined by the Global Leadership Initiative on Malnutrition and nutrition impact symptoms on the possibility of returning to work after treatment for head and neck cancer
Source: Support Care Cancer. 2023 Dec 22;32(1):55. doi: 10.1007/s00520-023-08252-x (PMC10746764; doi:10.1007/s00520-023-08252-x)
Supplement: Supplementary file 1 — Supplementary file1 (DOCX 22 KB) [file 520_2023_8252_MOESM1_ESM.docx]

**Appendix**

Table A. Difference in nutrition impact symptoms (NIS) over time measured by the Head and Neck Patient Symptom Checklist© (HNSC©). Results from the first part of the HNSC^©^ that shows NIS **intensity,** which assesses the frequency of the symptom during the last 3 days.

|  |  | **3 months** | | | **1 year** | | | **2 years** | | |  |
| --- | --- | --- | --- | --- | --- | --- | --- | --- | --- | --- | --- |
| **NIS** | Number of patients | Q1 | median | Q3 | Q1 | median | Q3 | Q1 | median | Q3 | p-value* |
| Pain | 129 | 1.0 | 2.0 | 3.0 | 1.0 | 1.0 | 2.0 | 1.0 | 1.0 | 2.0 | <0.001 |
| Anxious | 126 | 1.0 | 2.0 | 3.0 | 1.0 | 1.0 | 2.3 | 1.0 | 1.0 | 2.0 | 0.003 |
| Dry mouth | 127 | 3.0 | 4.0 | 4.0 | 2.0 | 3.0 | 4.0 | 2.0 | 3.0 | 4.0 | <0.001 |
| Loss of appetite | 127 | 1.0 | 2.0 | 4.0 | 1.0 | 1.0 | 2.0 | 1.0 | 1.0 | 2.0 | <0.001 |
| Constipation | 128 | 1.0 | 1.0 | 1.0 | 1.0 | 1.0 | 1.0 | 1.0 | 1.0 | 1.0 | 0.545 |
| Feeling full | 126 | 1.0 | 2.0 | 3.0 | 1.0 | 2.0 | 2.0 | 1.0 | 1.0 | 2.0 | <0.001 |
| Depressed | 128 | 1.0 | 1.0 | 2.0 | 1.0 | 1.0 | 2.0 | 1.0 | 1.0 | 2.0 | 0.141 |
| Thick saliva | 127 | 1.0 | 2.0 | 4.0 | 1.0 | 2.0 | 3.0 | 1.0 | 2.0 | 3.0 | 0.023 |
| Diarrhoea | 129 | 1.0 | 1.0 | 1.0 | 1.0 | 1.0 | 1.0 | 1.0 | 1.0 | 1.0 | 0.352 |
| Sore mouth | 129 | 1.0 | 1.0 | 2.0 | 1.0 | 1.0 | 2.0 | 1.0 | 1.0 | 2.0 | 0.036 |
| Lack of energy | 128 | 1.0 | 2.0 | 3.0 | 1.0 | 2.0 | 3.0 | 1.0 | 2.0 | 3.0 | <0.001 |
| Nausea | 129 | 1.0 | 1.0 | 1.0 | 1.0 | 1.0 | 1.0 | 1.0 | 1.0 | 1.0 | 0.414 |
| Diff. chewing | 127 | 1.0 | 1.0 | 2.0 | 1.0 | 1.0 | 2.0 | 1.0 | 1.0 | 1.0 | 0.064 |
| Smells bother me | 128 | 1.0 | 1.0 | 2.0 | 1.0 | 1.0 | 1.0 | 1.0 | 1.0 | 1.0 | 0.049 |
| Vomiting | 129 | 1.0 | 1.0 | 1.0 | 1.0 | 1.0 | 1.0 | 1.0 | 1.0 | 1.0 | 0.339 |
| Diff. swallowing | 126 | 1.0 | 2.0 | 2.0 | 1.0 | 1.0 | 2.0 | 1.0 | 1.0 | 2.0 | 0.018 |
| Taste changes | 129 | 2.0 | 3.0 | 4.0 | 1.0 | 2.0 | 4.0 | 1.0 | 2.0 | 3.0 | <0.001 |

*The Friedman test

Table B. Difference in nutrition impact symptoms (NIS) over time measured by the Head and Neck Patient Symptom Checklist© (HNSC©). Results from the second part of the HNSC^©^ that shows NIS **interference,** which assesses the interference of the symptom with oral intake.

|  |  | **3 months** | | | **1 year** | | | **2 years** | | |  |
| --- | --- | --- | --- | --- | --- | --- | --- | --- | --- | --- | --- |
| **NIS** | Number of patients^1^ | Q1 | median | Q3 | Q1 | median | Q3 | Q1 | median | Q3 | p-value* |
| Pain | 21 | 1.0 | 3.0 | 3.0 | 2.0 | 2.0 | 3.5 | 1.0 | 2.0 | 3.0 | 0.374 |
| Anxious | 35 | 1.0 | 2.0 | 3.0 | 1.0 | 2.0 | 2.0 | 1.0 | 1.0 | 2.0 | 0.330 |
| Dry mouth | 87 | 2.0 | 3.0 | 4.0 | 1.0 | 2.0 | 3.0 | 1.0 | 2.0 | 3.0 | <0.001 |
| Loss of appetite | 29 | 3.0 | 4.0 | 4.0 | 2.0 | 2.0 | 3.0 | 2.0 | 2.0 | 3.0 | <0.001 |
| Constipation | 4 | 2.0 | 2.0 | 2.75 | 1.0 | 1.5 | 2.0 | 2.0 | 2.0 | 3.5 | 0.150 |
| Feeling full | 39 | 2.0 | 2.0 | 3.0 | 2.0 | 2.0 | 3.0 | 1.0 | 2.0 | 2.0 | 0.013 |
| Depressed | 19 | 1.0 | 2.0 | 4.0 | 1.0 | 2.0 | 3.0 | 1.0 | 2.0 | 3.0 | 0.782 |
| Thick saliva | 59 | 1.0 | 2.0 | 3.0 | 1.0 | 1.0 | 3.0 | 1.0 | 2.0 | 2.0 | 0.002 |
| Diarrhoea | 6 | 1.0 | 2.0 | 2.0 | 1.0 | 3.0 | 3.0 | 1.0 | 1.5 | 3.25 | 0.819 |
| Sore mouth | 18 | 1.74 | 3.0 | 4.0 | 1.75 | 2.0 | 3.0 | 1.0 | 2.0 | 3.0 | 0.079 |
| Lack of energy | 46 | 1.0 | 1.5 | 3.0 | 1.0 | 1.0 | 2.0 | 1.0 | 1.0 | 2.0 | 0.024 |
| Nausea | 7 | 1.0 | 2.0 | 3.0 | 2.0 | 2.0 | 3.0 | 2.0 | 2.0 | 4.0 | 0.438 |
| Diff. chewing | 19 | 2.0 | 3.0 | 4.0 | 2.0 | 2.0 | 3.0 | 2.0 | 2.0 | 3.0 | 0.534 |
| Smells bother me | 8 | 1.25 | 2.5 | 3.75 | 2.0 | 3.0 | 3.0 | 1.0 | 2.0 | 3.0 | 0.211 |
| Vomiting | - | - | - | - | - | - | - | - | - | - | - |
| Diff. swallowing | 39 | 2.0 | 3.0 | 4.0 | 2.0 | 2.0 | 3.0 | 2.0 | 2.0 | 3.0 | 0.011 |
| Taste changes | 67 | 2.0 | 3.0 | 4.0 | 2.0 | 3.0 | 3.0 | 1.0 | 2.0 | 3.0 | <0.001 |

*The Friedman test

^1^Note: the low number of patients is due to the design of the HNSC. Patients who rated ‘1 = not at all’ as the intensity of one symptom, did not proceed to the second part regarding the interference of that symptom.
